# Editorial: Artificial intelligence solutions for decision making in robotics

**DOI:** 10.3389/frobt.2024.1389191

**Published:** 2024-03-12

**Authors:** Qasem Abu Al-Haija

**Affiliations:** Department of Cybersecurity, Faculty of Computer and Information Technology, Jordan University of Science and Technology, Irbid, Jordan

**Keywords:** artificial intelligence, decision making, automation and robotics, robotics, machine learning (ML), deep learning

## 1 Introduction

Imagine robots exhibiting cognitive behavior, maneuvering over unexpected barriers, and making instantaneous judgments in changing situations. Integrating Artificial Intelligence (AI) and robots makes this vision a reality. Artificial intelligence transforms robotic decision-making, enabling unparalleled autonomy, flexibility, and efficiency. This editorial delves into the empowerment of robots by AI, analyzes its influence on different sectors, and reveals the promising prospects.

### 1.1 Enhancing robots using artificial intelligence

Recall that robots with inflexible programming face challenges when confronted with unforeseen circumstances. Artificial intelligence revolutionizes the industry. It converts robots into intelligent beings who can perceive, think, and make real-time choices. Picture robots process data with machine learning, draw intricate conclusions using deep learning, and consistently adjust their actions via reinforcement learning. This enables robots to do various duties in manufacturing, healthcare, and other fields, functioning with an agility that was previously inconceivable.

### 1.2 Decision-making in dynamic environments

Picture a robot maneuvering through a chaotic production setting, working alongside people, and responding immediately to unexpected alterations. AI-driven robots perform very well in dynamic conditions. They use sensor data, computer vision, and probabilistic reasoning to understand their environment, predict future situations, and make decisions that maximize results. This agility is very beneficial in disaster response, where adaptation is essential.

### 1.3 Security, dependability, and credibility

Trust in decision-making algorithms is crucial in important applications such as driverless automobiles and surgical robots. AI improves safety through thorough testing, validation, and verification procedures. Explainable AI enhances trust by enabling people to comprehend robotic judgments, promoting openness and accountability. AI-powered robots may blend well with people, enhancing capabilities and reducing dangers.

### 1.4 Dealing with obstacles and moral deliberations

AI has great promise in robotics, but there are still obstacles to overcome. We must tackle algorithmic prejudice, data privacy issues, and the unexpected repercussions of autonomous decision-making. Researchers and practitioners aim to provide equitable, transparent, and comprehensive AI solutions, recognizing the many social effects of robots. Collaboration across several fields, such as robotics, AI, ethics, and politics, is essential to create ethical deployment guidelines.

### 1.5 Prospects and potential paths ahead

The potential for AI-driven decision-making in robots is tremendous. Advancements in AI algorithms will enable robots to do more complex jobs with accuracy and effectiveness. The applications range from smart agriculture to tailored healthcare, with limitless possibilities. Collaborating with academics, engineers, and policymakers may help us explore new possibilities in human-robot teamwork, advancing toward a more sustainable, inclusive, and technologically advanced future.

## 2 Published articles

In this Research Topic, “Artificial intelligence solutions for decision-making in robotics,” we have published four significant articles as follows.

### 2.1 Computational transcendence: responsibility and agency

This work by Deshmukh and Srinivasa explores the emergence of responsible behavior in non-cooperative games with autonomous agents through “computational transcendence” (CT). CT endows agents with an elastic sense of self, leading them to prioritize collective welfare over individual benefit. It is demonstrated in game theoretic scenarios like the Prisoners’ Dilemma and Collusion, and a multi-agent framework is proposed. CT adapts strategies based on interactions with other agents and outperforms reciprocity in promoting responsible autonomy. This framework holds promise for applications in various fields, motivating further research in responsible AI and computational modeling of the “sense of self.”

### 2.2 Development of an intelligent system based on metaverse learning for students with disabilities

The COVID-19 pandemic has increased reliance on the internet for work and study, integrating the metaverse into daily life. This work by Sghaier et al. proposes a simple, intelligent meta-learning system adaptable to various users. It involves creating a virtual learning environment in Open Simulator, linked to Moodle via Sloodle, for managing students and educational content. Performance evaluation showed robustness, with login times of 12 and 16 s for standalone and grid modes, respectively. Testing with 50 disabled learners revealed significant achievement differences between traditional and Sloodle-enabled test groups in a mathematics course, supporting the system’s effectiveness.

### 2.3 Autonomous detection and sorting of litter using deep learning and soft robotic grippers

This work by Almanzor et al. introduces LitterBot, an autonomous robotic system designed to detect, localize, and classify roadside litter. Utilizing a learning-based object detection algorithm trained on the TACO dataset, LitterBot identifies and categorizes garbage. It employs a modular manipulation framework with soft robotic grippers and real-time visual servoing for picking up objects of varying sizes and shapes in dynamic environments. Achieving over 80% success rates in picking and binning, LitterBot’s effectiveness was validated across various litter types and configurations. This demonstrates the successful deployment of a deep learning model in real-world applications through appropriate control framework design.

### 2.4 End-to-end Jordanian dialect speech-to-text self-supervised learning framework

This article by Safieh et al. addresses the scarcity of labeled speech data, particularly in Arabic dialects and low-resource languages, which is crucial for speech-to-text engines. It proposes an end-to-end, transformers-based model with a framework tailored for such languages, focusing on the Jordanian Arabic dialect. The framework integrates customized audio-to-text algorithms, facilitating data ingestion from various sources and expediting manual annotation via external ground truth. Employing noisy student training and self-supervised learning outperforms fine-tuned models by 5% in word error rate reduction. This work offers a Jordanian-spoken dataset and an efficient approach for low-resource languages, advancing applications like question-answering systems and intelligent robots.

## 3 Conclusion

AI is transforming the field of robotics by giving robots intelligence and autonomy. Artificial intelligence helps robots navigate intricate surroundings, engage with people, and efficiently execute jobs. Responsible development is essential to tackle ethical issues and secure a future where robots enhance human capacities for the common good. Through interdisciplinary collaboration and a focus on human-centered design, we can use the revolutionary potential of AI to create a future in which robots and humans collaborate to improve the world.

